# Legionella-like amebal pathogens--phylogenetic status and possible role in respiratory disease.

**DOI:** 10.3201/eid0203.960311

**Published:** 1996

**Authors:** A Adeleke, J Pruckler, R Benson, T Rowbotham, M Halablab, B Fields

**Affiliations:** Kings' College, London, UK.


### *Legionella*-Like Amebal Pathogens––Phylogenetic Status and Possible Role in Respiratory Disease

*Legionella*-like amebal pathogens (LLAPs) are nonculturable, protozoonotic, gram-negative bacilli. They were named LLAPs because of their ability to infect and multiply intracellularly within amebae (Figure 1) in the same way that legionellae infect and multiply in protozoa and human alveolar macrophages (1-3). However, unlike other known legionellae, LLAPs do not grow routinely on buffered charcoal yeast extract agar (BCYE), or on any other known bacteriologic media.

The first known LLAP was isolated from Polish soil by Drozanski in 1954 (4). Until 1991 when it was described and given the name *Sarcobium lyticum* (5), it was simply referred to as an obligate intracellular bacterial parasite of free-living amebae (6, 7). The next isolation of an LLAP was in England more than 20 years later. Since then, LLAPs have been commonly isolated from various sources and found to infect a variety of amebae (Table 1). Because one LLAP cannot be differentiated from another, each strain is given the name LLAP suffixed by a designated number. Except for *S. lyticum*, LLAP-1, LLAP-5, and LLAP-3, which was a clinical isolate, all LLAPs were originally isolated from sources associated with confirmed cases or outbreaks of Legionnaires' disease (Table 1).

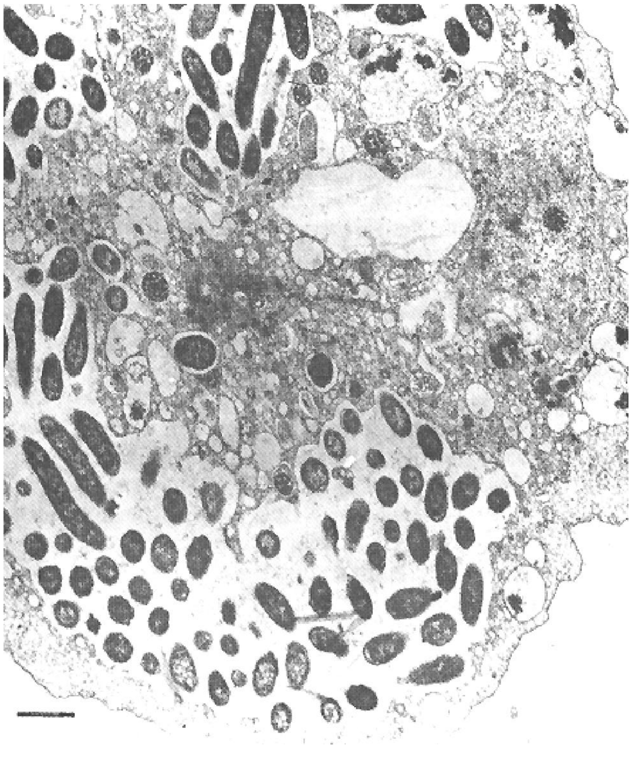

Figure 1. Transmission electron micrograph of *Sarcobium lyticum* within *Acanthamoeba castellanii*. Bar = 1 µm. (Reproduced with permisssion from reference 7).

Amebae are natural hosts for legionellae in the environment (2) and have been used to isolate legionellae from clinical samples (8). The relationship between these organisms is unique in that amebae, which generally use other bacteria as food, are parasitized by legionellae. The mechanism by which legionellae multiply and evade host defenses in amebae simulates the mechanism of infection in humans (9,10). The ability of *Legionella pneumophila* to infect epithelial cells in vitro is also enhanced by prior cultivation of the bacteria in amebae (11). This suggests that there may be a connection between the pathogenicity of legionellae for mammalian cells and animal models and their pathogenicity for amebae.

Whereas members of the genus *Legionella*, particularly *L. pneumophila*, are recognized as important etiologic agents of pneumonia (12), little is known about the clinical relevance of LLAPs; nevertheless, some evidence suggests that they may be an unrecognized and possibly significant cause of respiratory disease. LLAPs' inability to grow or multiply in the absence of amebae makes it difficult to isolate or identify them in clinical samples by conventional techniques. No serologic reagents are currently available for the detection or identification of LLAPs in clinical specimens. It is particularly noteworthy that one LLAP strain was originally isolated by amebal enrichment of a sputum specimen from a patient with pneumonia (13). Clinical samples from the patient were culture and serologically negative for *L. pneumophila* serogroups 1 through 6. However, incubation of the sputum sample with *Acanthamoeba polyphaga* resulted in numerous bacteria-infected amebae. The patient demonstrated a fourfold rise in antibody titer to the bacteria from the infected amebae. Even though the bacteria, now known as LLAP-3, could not be recovered on BCYE, the patient was treated for *Legionella* infection and recovered (13). Attempts to culture LLAP-3 on BCYE or on any other artificial media have been unsuccessful.

Since the discovery of LLAP-3, there has been further serologic evidence for the involvement of LLAPs in pneumonia. Between 1989 and 1993, the Public Health Laboratory (Leeds, UK) routinely

Table 1. Source of *Legionella*-like amebal pathogen (LLAP)

| Strain | Host | Temp(°C) | Original source | Year |
|---|---|---|---|---|
| *S. lyticum* | AP** | 35 | Soil isolate[a] | 1954 |
| LLAP-1 | AP | 30[N35] | Tank of portable water well | 1981 |
| LLAP-2 | AP** | 35 | Garage steam cleaning pit | 1986 |
| LLAP-3 | AP1 | 35 | Sputum from pneumonia patient | 1986 |
| LLAP-4 | AP | 30[N35] | Hospital whirlpool bath | 1986 |
| LLAP-5[LST] | AP | 30 | Nursing home plant spray | 1988 |
| LLAP-6 | AP** | 35 | Factory liquefier tower | 1988 |
| LLAP-7 | AP** | 35 | Hotel whirlpool spa | 1991 |
| LLAP-8 | HV[NAP] | 35 | Hospital shower | 1990 |
| LLAP-9 | AP** | 35 | Factory cooling water | 1992 |
| LLAP-10 | AP | 35 | Ship air-conditioning system | 1994 |
| LLAP-11 | AP | 30 | Factory cooling tower | 1993 |
| LLAP-12 | AP | 30 | Factory cooling system | 1994 |

a = first LLAP isolate. AP = infected *Acanthamoeba polyphaga* isolated from original material. AP1 = isolated after coculitivation of sample with *A. polyphaga*. HV = *Hartmannella vermiformis* isolated from original material. ** = also multiplies in *H. vermiformis*. NAP = does not infect *A. polyphaga*. LST = lost strain. N35 = does not multiply at 35°C.

screened sera from patients with suspected Legionnaires' disease for antibodies to LLAP-3 and found more than 10 cases with appropriately timed rising titers and several convalescent-phase sera. A few cases of respiratory disease with rising titers to LLAP-1 and little activity to LLAP-3 were also found (13).

In a study to assess the role of LLAPs in community-acquired pneumonia, 19% of 500 hospitalized patients with pneumonia of unidentified origin demonstrated a ≥ fourfold rise in antibody titer to 1:128 to at least one of nine LLAPs tested (14). These patients had no evidence of infection with known *Legionella* species (15). Cross-reactivity or nonspecific antibody rises may have accounted for some of the cases. Nevertheless, these findings are particularly important since no etiologic agent is identified for an estimated 50% of the 500,000 adult pneumonia cases occurring annually in the United States (16-18).

With the exception of *S. lyticum*, LLAPs have not been named or classified because the inability to cultivate them on laboratory media has limited their genetic and biochemical characterization. In this study, LLAPs were cocultured with *Hartmannella vermiformis* and *Acanthamoeba polyphaga,* depending on the most suitable host for each LLAP. Samples were cultured on blood agar and BCYE without cysteine to rule out bacterial contaminants. Bacteria were separated from amebae by differential centrifugation of cocultures before isolation of genomic DNA for ribosomal DNA sequencing.

The genes that code for rRNA in bacteria are highly conserved and can be used to establish the phylogenetic relationship between organisms (19). Previous 16S rDNA analysis of *S. lyticum* (20) and LLAP-3 (21), showed that they are closely related (91.6% to 95.8% rRNA similarity) to members of the genus *Legionella*. It was, therefore, suggested that LLAPs may have been responsible for the Gen-Probe positive specimens from patients with pneumonic illness, for whom conventional culture and serologic tests for legionellae were negative (13,22,23). The Gen-Probe system is a rapid diagnostic test for legionellae that uses a DNA probe complementary to the rRNAs of *Legionella* species (23,24).

To establish the degree of relatedness of all known LLAPs to each other and to other members of the family *Legionellaceae,* we recently amplified the 16S rRNA gene from five LLAP strains (LLAP-1, LLAP-2, LLAP-7, LLAP-8, and LLAP-9), using primers specific for Eubacteria. The amplified DNA was sequenced and compared with other bacterial sequences (including those of all *Legionella* species and LLAPs) held by GenBank/EMBL. Analysis of the 16S rRNA gene sequences showed that all 12 LLAPs in this study are closely related (91.2% to 97.0% similarity over 1303 bases of 16S rRNA sequence) to members of the genus *Legionella.* This is consistent with the 16S rRNA sequence identity (90.2% to 99.1% over 1303 bases) that exists between the 39 validly described members of the genus.[1]

Phylogenetic analysis showed that all the LLAPs in this study form a coherent cluster with other

[1]Genomic DNA was isolated by standard methods and extracted with phenol/chloroform. A 1400-bp 16S rDNA segment was amplified by using primers specific for Eubacteria and based on a consensus of *Legionella* 16S rRNA sequences. Each strain was sequenced at least 3 times to ascertain accuracy of obtained results. 16S rRNA sequences for all *Legionella* species, those of some previously sequenced LLAPs and *Coxiella burnetii* were obtained from GenBank/EMBL and aligned with sequences generated in this study, using the GCG sequence analysis package (version 8.0). Comparisons were performed at NCBI using the BLAST network service (32). Ambiguous and hypervariable regions were removed before phylogenetic analysis, which was carried out on 53 strains for 1303 nucleotides by the neighbor-joining method from the Phylogeny Inference Package (Phylip) (33), version 3.5. Multiple datasets (X100) were analyzed, and different distance models were compared to ensure reliability. Some of the 16S rRNA sequences included in the data analysis are of unpublished and undescribed stains.

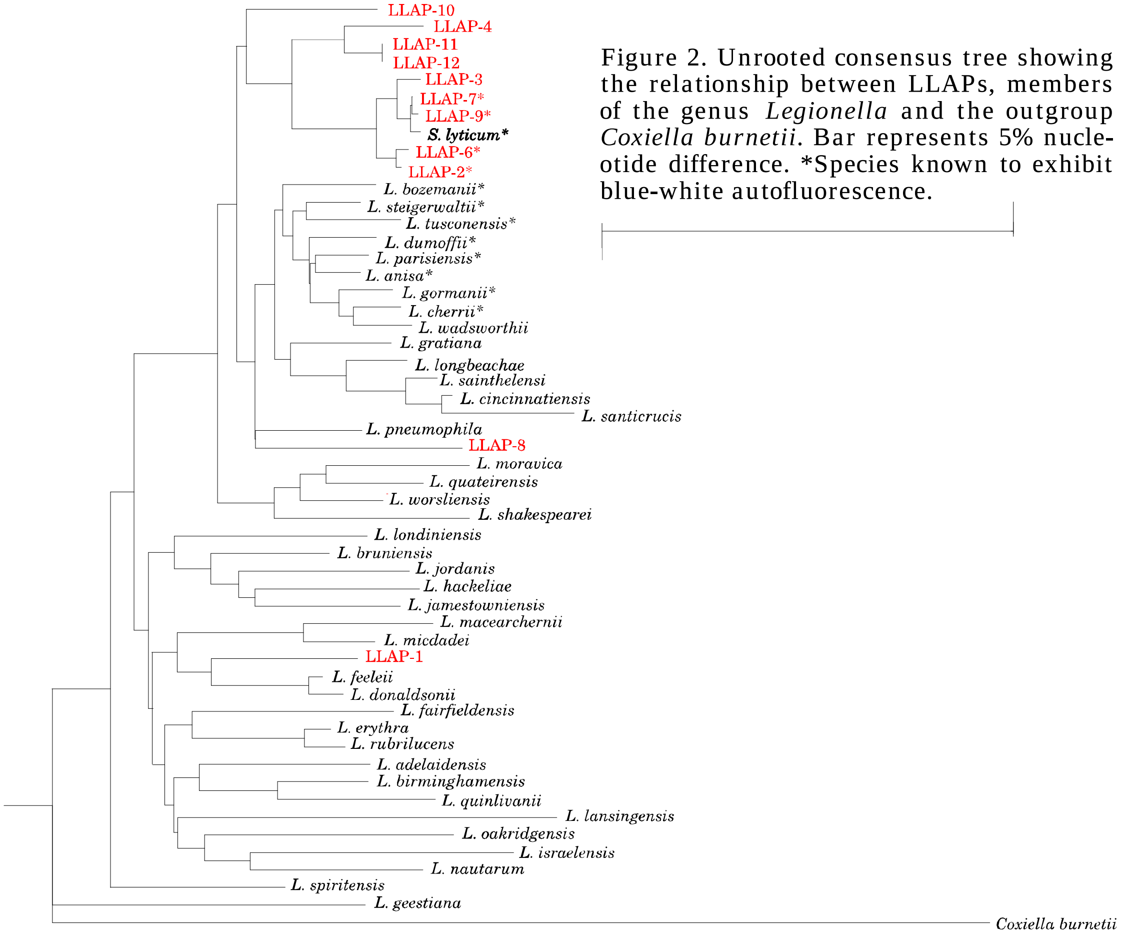

Figure 2. Unrooted consensus tree showing the relationship between LLAPs, members of the genus *Legionella* and the outgroup *Coxiella burnetii*. Bar represents 5% nucleotide difference. *Species known to exhibit blue-white autofluorescence.

members of the family *Legionellaceae* (Figure 2). LLAP-1, LLAP-8, and LLAP-10 were grouped in distinctly different clades, whereas LLAP-2, LLAP-3, LLAP-6, LLAP-7, LLAP-9, and *S. lyticum* formed a single well-defined clade, most closely related ($\geq$96.9% similarity) to another clade consisting of LLAP-4, LLAP-11, and LLAP-12 (Figure 2). LLAP-2, LLAP-3, LLAP-6, LLAP-7, LLAP-9, and *S. lyticum* shared a 16S rRNA sequence similarity of $\geq$98.9%. LLAP-11 and LLAP-12 had 16S rRNA sequence homology of 100%. Overall 16S rRNA sequence similarity values from some *Legionella* species, all the LLAPs in this study, and *Coxiella burnetii* are shown in Table 2. These results suggest that these 12 LLAP strains may represent up to five new species of *Legionella* and their various subtypes on the basis of a minimum sequence identity of 97.0% between strains of the same species (Table 3). However, they may include new subtypes of previously described species. It is also possible that these strains represent more than five species because, even though 16S rRNA sequence identity can be used routinely to distinguish and establish relationships between genera and well-resolved species, it may not be sufficient to guarantee species identity (25,26). For example, in this study, several *Legionella* species had sequence similarities exceeding 98.0%. *L. cherrii* and *L. wadsworthii* shared a sequence similarity of 98.7%. *L. rubrilucens* and *L. erythra* had a sequence identity of 99.1%. *L. sainthelensi* and *L. cincinnatiensis* were likewise identical by 99.1%. Furthermore, bacterial strains exhibiting more than

*Footnote 1 continued*

Accession numbers for sequences included in this study are as follows: LLAP-1 NL, LLAP-2 U44909, LLAP-3 X60080, LLAP-4 X97357, LLAP-6 X97357, LLAP-7 U44910, LLAP-8 NL, LLAP-9 U44911, LLAP-10 X97363, LLAP-11 X97362, LLAP-12 X97366. *L. adelaidensis Z49716, L.anisa X733394, L. birminghamensis Z49717, L. brunensis X73403, L. cherrii X73404, L. cincinnatiensis X73407, Coxiella burnetti M21291, L. donaldsonii Z49724, L.dumoffii X73405, L.erythra Z32638, L. fairfieldensis Z49722, L. feelii X73395, L. geestiana Z49723, L. gormanii Z32639, L. gratiana Z49725, L. hackeliae M36028, L. israelensis X73408, L. jamestowniensis X73409, L. jordanis X73396, L. lansingensis Z49727, L. londiniensis Z49728, L. longbeachae M36029, L. maceachernii X60081, L. micdadei M36032, L. moravica Z49729, L. nautarum Z49730, L. oakridgensis X73397, L. parisiensis Z49731, L. pneumophila M59157, L. quateirensis Z49732, L. worsliensis Z49739, L. quinlivanii Z49733, L. rubrilucens X73398, L. santicrucis Z49735, Sarcobium lyticum X66835, L. shakespearei Z49736, L. spiritensis M36030, L. steigerwaltii X73400, L. sainthelensi Z49734, L. tusconensis Z32644, L. wadsworthii X73401.* NL = accession number not yet allocated.

Table. 2. 16S rRNA similarities (%) between members of the genus Legionella and different LLAP strains.

| L1 | L2 | L3 | L4 | SL | L6 | L7 | L8 | L9 | L10 | L11 | L12 | Lpn | Lfl | Lgr | Lts | Lpr | Lst | Ldm | Lwd | Cox |
|---|---|---|---|---|---|---|---|---|---|---|---|---|---|---|---|---|---|---|---|---|
| L1 | 94.1 | 93.6 | 93.9 | 93.7 | 94.0 | 93.7 | 94.1 | 93.7 | 94.4 | 94.1 | 94.1 | 93.8 | 96.6 | 93.7 | 94.0 | 93.9 | 94.3 | 94.1 | 94.0 | 85.4 |
| L2 | | 98.9 | 96.9 | 99.2 | 99.8 | 99.4 | 95.4 | 99.3 | 96.6 | 97.4 | 97.4 | 95.8 | 94.5 | 96.2 | 96.3 | 96.3 | 96.7 | 96.8 | 96.3 | 85.6 |
| L3 | | | 96.9 | 99.4 | 99.0 | 99.5 | 94.9 | 99.5 | 96.3 | 97.0 | 97.0 | 95.8 | 93.9 | 95.7 | 96.2 | 96.1 | 96.5 | 96.4 | 96.0 | 85.6 |
| L4 | | | | 97.0 | 97.0 | 97.2 | 95.1 | 97.1 | 96.0 | 98.5 | 98.5 | 95.5 | 93.4 | 95.5 | 95.9 | 95.9 | 96.0 | 95.9 | 96.0 | 85.2 |
| SL | | | | | 99.1 | 99.9 | 94.9 | 99.8 | 96.3 | 97.2 | 97.2 | 95.8 | 94.1 | 95.5 | 96.1 | 95.9 | 96.4 | 96.3 | 96.0 | 85.6 |
| L6 | | | | | | 99.3 | 95.1 | 99.2 | 96.8 | 97.3 | 97.3 | 95.6 | 94.4 | 96.1 | 96.4 | 96.3 | 96.5 | 96.5 | 96.1 | 85.5 |
| L7 | | | | | | | 95.0 | 99.9 | 96.5 | 97.4 | 97.4 | 95.9 | 94.1 | 95.7 | 96.3 | 96.0 | 96.6 | 96.5 | 96.1 | 85.8 |
| L8 | | | | | | | | 94.9 | 94.9 | 95.7 | 95.7 | 96.1 | 95.1 | 95.9 | 95.9 | 95.9 | 95.8 | 96.1 | 95.4 | 85.5 |
| L9 | | | | | | | | | 96.4 | 97.3 | 97.3 | 95.8 | 94.0 | 95.6 | 96.2 | 95.9 | 96.5 | 96.4 | 96.0 | 85.8 |
| L10 | | | | | | | | | | 96.3 | 96.3 | 96.7 | 94.4 | 96.3 | 95.9 | 97.0 | 96.3 | 96.4 | 96.6 | 85.9 |
| L11 | | | | | | | | | | | 100.0 | 96.3 | 94.0 | 96.3 | 97.0 | 96.7 | 96.9 | 96.5 | 96.5 | 85.0 |
| L12 | | | | | | | | | | | | 96.3 | 94.0 | 96.3 | 97.0 | 96.7 | 96.9 | 96.5 | 96.5 | 85.0 |
| Lpn | | | | | | | | | | | | | 94.3 | 96.8 | 96.9 | 97.3 | 97.5 | 97.0 | 96.8 | 85.5 |
| Lfl | | | | | | | | | | | | | | 94.4 | 94.9 | 95.0 | 95.2 | 95.4 | 95.1 | 86.2 |
| Lgr | | | | | | | | | | | | | | | 97.1 | 97.2 | 97.4 | 97.6 | 96.4 | 85.5 |
| Lts | | | | | | | | | | | | | | | | 97.8 | 98.1 | 97.3 | 96.8 | 85.8 |
| Lpr | | | | | | | | | | | | | | | | | 98.1 | 98.4 | 98.0 | 86.3 |
| Lst | | | | | | | | | | | | | | | | | | 98.4 | 97.6 | 85.7 |
| Ldm | | | | | | | | | | | | | | | | | | | 98.0 | 86.1 |
| Lwd | | | | | | | | | | | | | | | | | | | | 85.8 |

L. LLAP, Lpn. *L. pneumophila*, Lfl. *L. feeleii*, Lgr. *L. gratiana*, Lts. *L. tusconensis*, Lpr. *L. parisiensis*, Lst. *L steigerwaltii*, Ldm. *L. dumoffii*, Lwd. *L. wadsworthii*, Cox. *Coxiella burnetii*.

exhibiting more than 99.5% rRNA sequence identity may belong to different species by current standards for DNA-DNA hybridization (70% DNA reassociation) (25). DNA hybridization studies are, therefore, needed to determine species identity.

Preliminary fatty acid analysis showed that these LLAPs have branched fatty acids, characteristic of members of the genus *Legionella*. The gas liquid chromatography fatty acid profiles obtained, however, appeared to be distinct from those of other known legionellae (data not shown).

LLAP-2, LLAP-3, LLAP-7, and LLAP-9 reacted positively with antiserum prepared against *S. lyticum*. Fluorescent antibody tests also showed some LLAPs to react positively with the polyvalent *Legionella* antiserum contained in the Remel *Legionella* Poly-ID Test Kit.[2]

Several attempts have been made to culture LLAPs on conventional *Legionella* media, BCYE. *S. lyticum* could occasionally be recovered from a coculture with amebae on alanine supplemented BCYE (27). We attempted to grow 12 LLAP strains on regular BCYE and BCYE supplemented with various amino acids, including alanine, and have recently succeeded in cultivating LLAP-6, LLAP-7, LLAP-9, and LLAP-10 on bacteriologic media for the first time. All colonies had the characteristic cut-glass appearance associated with legionellae. LLAP-1 had previously been cultured on BCYE supplemented with sodium selenate (13,28). In this study, LLAP-1 was successfully cultured on regular BCYE and BCYE supplemented with alanine in the absence of sodium selenate. *S. lyticum*, LLAP-1, LLAP-6, LLAP-7, LLAP-9, and LLAP-10 had a cysteine requirement for growth. Other factors influencing growth are yet to be determined, but sodium selenate and alanine may not necessarily be required for growth. Cultivation of LLAPs on bacteriologic media still cannot be considered a routine process because recovery is not always possible, and extremely low numbers of the plated bacteria produce colonies.

Some species of *Legionell*a are known to produce colonies that autofluoresce on exposure to long-wave ultraviolet (UV) light. This facet of their colonial morphology often assists in identifying and speciating isolates. LLAP-2, LLAP-6, LLAP-7, LLAP-9, and *S. lyticum* exhibited blue-white autofluorescence when exposed to long-wave UV light while LLAP-1 and LLAP-10 did not autofluoresce. This is consistent with the clustering pattern (Figure 2) where the blue-white fluorescent LLAPs formed a well-defined clade.

On the basis of these results, we propose that all 12 *Legionella*-like amebal pathogens be included in the genus *Legionella* as *Legionella* species, with

[2]The Remel Poly-ID kit used in this study is designed to identify 22 species and 31 serogroups of *Legionella* (34,35).

Table 3. Possible number of species represented by *Legionella*-like amebal pathogens (LLAP)

| Species 1 | Species 2 | Species 3 | Species 4 | Species 5 |
|---|---|---|---|---|
| LLAP-2 | LLAP-1 | LLAP-8 | LLAP-10 | LLAP-4 |
| LLAP-3 | | | | LLAP-11 |
| LLAP-6 | | | | LLAP-12 |
| LLAP-7 | | | | |
| LLAP-9 | | | | |
| *S. lyticum* | | | | |

Note. The table above includes the minimum number of species that may be represented by the 12 LLAPs in this study.

strain specifications. A proposal was recently made for the transfer of the species *S. lyticum* to the genus *Legionella* as *L. lytica* comb. nov. (29). It appeared that *L. lytica* was being proposed for LLAPs. However, our study suggests that LLAPs may represent a minimum of five species. Consequently, the species name *L. lytica*, though appropriate for the strain formerly classified as *S. lyticum*, cannot be applied to all LLAPs.

The genus *Legionella* is defined in *Bergey's Manual of Determinative Bacteriology* as follows:

> Rods measuring 0.3-0.9 x 2.0-20.0 µm or more. Do not form endospores or microcysts and are not encapsulated. Not acid fast. Cells stain gram negative. Motile by one, two, or more straight or curved polar or lateral flagella; non motile strains are occasionally seen. Aerobic. L-Cysteine hydrochloride and iron salts are required for growth. Oxidase test is negative or weakly positive. Nitrates are not reduced. Urease negative. Gelatin is liquified. Branched chain fatty acids predominate cell wall. Chemoorganotrophic, using amino acids as carbon and energy sources. Carbohydrates are neither fermented nor oxidized. Isolated from surface water, mud, and from thermally polluted lakes and streams. There is no known soil or animal source. pathogenic for humans, causing pneumonia (Legionnaires' disease) or a mild, febrile disease (Pontiac fever) (30,31).

The description in *Bergey's Manual* should be amended to include the following statement: "Some legionellae appear to be primarily obligate intracellular parasites of amebae that exhibit little or no growth on current laboratory media."

Further characterization is needed to classify each LLAP strain to species level and assess the need for additional amendments to the present description of the genus *Legionella*. Appropriate diagnostic techniques are needed to determine the clinical relevance of LLAPs, and particularly their possible role in respiratory disease.

### Acknowledgements

We are very grateful to Drs. Anne Whitney, Chi-Cheng Luo, and Catherine Bender for valuable assistance with analysis of sequence data. The picture of *S. lyticum* within *A. castellanii* was reprinted with permission from Prof. W. Drozanski of the Institute of Microbiology, Maria Curie-Sklodowska University, Poland.

Some of these data were presented at the 96th General Meeting of the American Society for Microbiology in May 1996.

**Adenike Adeleke, M.S.,* Janet Pruckler,† Robert Benson, M.S.,† Timothy Rowbotham, Ph.D.,‡ Mahmoud Halablab, Ph.D.,* Barry Fields, Ph.D.†**

*Kings' College, London, UK; †Centers for Disease Control and Prevention, Atlanta, Georgia, USA; ‡Public Health Laboratory, Leeds, UK

### References

1. Rowbotham TJ. Preliminary report on the pathogenicity of *Legionella pneumophila* for fresh water and soil amoebae. J Clin Pathol 1980;33:1179-83.
2. Rowbotham TJ. Current views on the relationship between amoebae, legionellae and man. Isr J Med Sci 1986;22:678-89.
3. Chandler FW, Hicklin MD, Blackmon JA. Demonstration of the agent of Legionnaires' disease in tissue. N Engl J Med 1977;297:1218-20.
4. Drozanski W. Fatal bacterial infection in soil amoebae. Acta Microbiol Pol 1956;5:315-7.
5. Drozanski W. *Sarcobium lyticum* gen. non. sp. nov., an obligate intracellular bacterial parasite of small free living amoebae. Int. J Syst Bacteriol 1991;41:82-7.
6. Drozanski W, Chmielewski T. Electron microscope studies of *Acanthamoeba castellanii* infected with obligate intracellular bacterial parasite. Acta Microbiol Pol 1979;128:123-33.
7. Drozanski W, Madra L, Chmielewski T, Schlecht S, Golecki JR. Evidence for the presence of N-unsubstituted glucosamine residues in the peptidoglycan *Sarcobium cytolyticum*—an intacellular bacterial parasite of amoebae. System Appl Microbiol 1990;13:220-6.
8. Rowbotham TJ. Isolation of *Legionella pneumophila* from clinical specimens via amoebae, and the interaction of those and other isolates with amoebae. J Clin Pathol 1983;36:978-86.
9. Cianciotto NP, Fields BS. *Legionella pneumophila mip* gene potentiates intracellular infection of protozoa and human macrophages. Proc Natl Acad Sci USA 1992; 89:5188-91.
10. Fields BS, Barbaree JM, Shotts EB JR, Feeley JC, Morrill WE, Sanden GN, et al. Comparison of guinea pig and protozoan models for determining virulence of *Legion-ella* species. Infect Immun 1986;53:553-9.
11. Cirillo JD, Falkow S, Tompkins LS. Growth of *Legionella pneumophila* in *Acanthamoeba castellanni* enhances invasion. Infect Immun 1994;62:3254-61.
12. McDade JE, Shepard CC, Fraser DW, Tsai TR, Redus MA, Dowdle WR, et al. Legionnaires' disease: isolation of a bacterium and demonstration of its role in other respiratory disease. N Engl J Med 1977;297:1197-203.

13. Rowbotham T J. *Legionella*-like amoebal pathogens. In: Barbaree JM, Breiman RF, Dufour AP. *Legionella*: current status and emerging perspectives. Washington DC. American Society for Microbiology, 1993: 137-40.
14. Benson RF, Drozanski WJ, Rowbotham TJ, Fields BS, Bialkowska I, Losos D, et al. Serologic evidence of infection with 9 *Legionella*-like amoebal pathogens in pneumonia patients. Proceedings of the 95th Annual General Meeting of the American Society for Microbiology, Washington DC, USA. 1995: Abstract C-200.
15. Butler JC, Benson RF, Drozanski WJ, Rowbotham TJ, Bialkowska I, Losos D, et al. Pneumonia associated with *Legionella*-like amoebal pathogen (LLAP) infection: patient characteristics and clinical manifestations. Proceedings of the 35th Interscience Conference on Antimicrobial Agents and Chemotherapy. American Society for Microbiology, San Francisco, California, USA. 1995: Abstract K-61.
16. Garibaldi RA. Epidemiology of community acquired respiratory tract infections in adults: incidence, etiology, and impact. Am J Med 1985;78:32-7.
17. Fass RJ. Aetiology and treatment of community-acquired pneumonia in adults: an historical perspective. J Antimicrob Chemother 1993;32;17-27.
18. Kerttula Y, Leinonen M, Koskela M, Makela PH. The aetiology of pneumonia. Application of bacterial serology and basic laboratory methods. J Infect 1987;14:21-30.
19. Woese CR. Bacterial evolution. Microbiol Rev 1987;51:221-71.
20. Springer N, Ludwig W, Drozanski W, Amann R, Schleifer KH. The phylogenetic status of *Sarcobium lyticum*, an obligate intracellular bacterial parasite of small amoebae. FEMS Microbiol Lett 1992;96:199-202.
21. Fry NK, Rowbotham TJ, Saunders NA, Embly TM. Direct amplification and sequencing of the 16S ribosomal DNA of an intacellular *Legionella* species recovered by amoebal enrichment from the sputum of a patient with pneumonia. FEMS Microbiol Lett 1991;83:165-8.
22. Laussucq S, Schuster D, Alexander WJ, Thacker WL, Wilkinson HW, Spika JS. False positive DNA probe test for *Legionella* species associated with a cluster of respiratory illnesses. J Clin Microbiol 1988;26:1442-4.
23. Doebbling BN, Bale MJ, Koontz FP, Helms CM, Wenzel RP, Pfaller MA. Prospective evaluation of the Gen-Probe assay for detection of legionellae in respiratory specimens. Eur J Clin Microbiol Infect Dis 1988;7:748-52.
24. Edelstein PH, Bryan RN, Enns RK, Kohne DE, Kacian DL. Retrospective study of Gen-Probe Rapid Diagnostic System for detection of legionellae in frozen clinical respiratory tract samples. J Clin Microbiol 1987;25:1022-6.
25. Fox GE, Wisotzkey JD, Jurtshuk P JR. How close is close; 16S rRNA sequence identity may not be sufficient to guarantee species identity. Int J Syst Bacteriol 1992;42:166-70.
26. Stackebrandt E, Goebel BM. Taxonomic note: a place for DNA-DNA reassociation and 16S rRNA sequence analysis in the present species definition in bacteriology. Int J Syst Bacteriol 1994;44:846-9.
27. Giles DL, Fields BS, Newsome AL, Drozanski WJ. Cultivation of *Sarcobium lyticum* on artificial medium. Proceedings of the 96th Annual General Meeting of the American Society for Microbiology, Washington DC, 1995: Abstract Q-447.
28. Smalley DL, Jaquess PA, Layne JS. Selenium-enriched medium for *Legionella pneumophila*. J Clin Microbiol 1980;12:32-4.
29. Hookey JV, Saunders NA, Fry NK, Birtles RJ, Harrison TJ. Phylogeny of *Legionellaceae* based on small-subunit ribosomal DNA sequences and proposal of *Legionella lytica* comb. nov. for *Legionella*-like amoebal pathogens. Int J Syst Bacteriol 1996;46:526-31.
30. Holt JG, Krieg NR, Sneath PHA, Staley JT, Williams ST. Bergey's manual of determinative bacteriology, Baltimore: Williams & Wilkins. 1994:86.
31. Brenner DJ, Feeley JC, Weaver RE. Family VII. Genus I. *Legionella* Brenner, Steigerwalt and McDade 1979. 658[AL]. In: Krieg NR, Holt JG editors: Bergey's manual of systematic bacteriology, 1. Baltimore: Williams & Wilkins, 1984:279-88.
32. Atschul SF, Gish W, Miller W, Myers EW, Lipman DJ. Basic local alignment search tool. J Mol Biol 1990;215:403-10.
33. Felsenstein J. PHYLIP-Phylogeny inference package. Cladistics 1989;5:164-6.
34. Thacker WL, Plikaytis BB, Wilkinson HW. Identification of 22 *Legionella* species and 33 serogroups with the slide agglutination test. J Clin Microbiol 1985;21:779-82.
35. Brown SL, Bibb WF, McKinney RM. Retrospective examination of lung tissue specimens for the presence of *Legionella* organisms: comparison of an indirect fluorescent antibody system with direct fluorescent antibody testing. J Clin Microbiol 1984;19:468-72.
